# Adrenal Insufficiency and Thyrotoxicosis Following Combined Immune Checkpoint Inhibitor Use: A Case Report and Literature Review

**DOI:** 10.7759/cureus.60850

**Published:** 2024-05-22

**Authors:** Kaoruko Fukushima, Saori Kitayama, Manami Sazuka, Remi Kodera, Kazuhito Oba, Kenji Toyoshima, Yuko Chiba, Hiroshi Yamamoto, Atsushi Araki, Yoshiaki Tamura

**Affiliations:** 1 Department of Diabetes, Metabolism, and Endocrinology, Tokyo Metropolitan Institute for Geriatrics and Gerontology, Tokyo, JPN; 2 Department of Respiratory Medicine, Tokyo Metropolitan Institute for Geriatrics and Gerontology, Tokyo, JPN

**Keywords:** combined nivolumab and ipilimumab therapy, thyrotoxicosis, adrenal insufficiency, immune-related adverse event (irae), immune checkpoint inhibitor (ici)

## Abstract

Destructive thyroiditis and secondary adrenal insufficiency are major endocrinological immune-related adverse events of immune checkpoint inhibitors (ICIs). However, the timing at which each event occurs most frequently after drug administration varies, and cases where multiple events occur simultaneously are rare. We encountered a patient who concurrently suffered from thyrotoxicosis and adrenal insufficiency.

An 80-year-old woman with a history of type 2 diabetes mellitus (DM) was diagnosed with stage IVA squamous cell carcinoma of the lungs. Treatment with a combination of nivolumab and ipilimumab was initiated. Although she tested positive for thyroglobulin antibody and transient subclinical hyperthyroidism was observed after two courses, treatment with ICIs was continued. Four months later, treatment was discontinued due to drug-induced lung disease. One month after the last administration, the patient became unconscious and was admitted to another hospital, diagnosed with diabetic ketoacidosis, urinary tract infection, and sepsis. After acute-phase treatment, she was transferred to our hospital due to persistent fever and tachycardia. Thyrotoxicosis and adrenal insufficiency were observed, with high levels of free thyroxine, low thyroid-stimulating hormone (TSH), and cortisol levels. Treatment with extracellular fluids, potassium iodide, beta-blockers, and hydrocortisone was initiated, and the patient’s condition improved. No other pituitary hormone deficiencies were observed. She was diagnosed with painless thyroiditis and secondary adrenal insufficiency based on the positive thyroglobulin antibody, negative TSH receptor antibody, decreased Doppler flow in thyroid ultrasonography, low adrenocorticotrophic hormone (ACTH), and low response of ACTH and cortisol to corticotropin-releasing hormone loading test. MRI revealed no abnormalities. We report a case of thyrotoxicosis and secondary adrenal insufficiency five months after the first administration of nivolumab and ipilimumab. Careful follow-up and early detection of endocrine disorders are critical in patients treated with a combination of ICIs.

## Introduction

Immune checkpoint inhibitors (ICIs) have become an essential treatment for multiple types of cancer, including melanoma, lung cancer, and renal cancer [1]. By suppressing programmed cell death-1 (PD-1), programmed death ligand 1 (PD-L1), and cytotoxic T-lymphocyte antigen-4 (CTLA-4) receptors, ICIs enhance the immune response against cancer cells [1]. However, ICIs also activate autoantigen-responsive T cells, which can lead to immune-related adverse events (irAEs). Such irAEs can affect various tissues, including endocrine organs. Endocrine irAEs reported include pituitary disorders, thyroid dysfunction, adrenal insufficiency, hypothyroidism, and type 1 diabetes mellitus (DM) [2]. ICI combination therapy has been found to be more likely to cause adverse endocrine events than monotherapy [3]. Additionally, the timing of each adverse event varies. The peak onset of thyroid dysfunction is observed at six to seven weeks [4], thyrotoxicosis occurs two weeks after the administration of ICI combination [5], and hypophysitis typically appears at 10 weeks [4, 6]; thus, simultaneous onset of multiple events is rare. Here, we describe a case of simultaneous onset of ICI-induced thyrotoxicosis and adrenal insufficiency following the administration of combined nivolumab and ipilimumab.

## Case presentation

An 80-year-old woman was found to have a 25 mm tumor in the lower lobe of the right lung on abdominal CT. She also had type 2 DM, hypertension, and hyperlipidemia, and had been treated with miglitol, repaglinide, vildagliptin, metformin hydrochloride, olmesartan medoxomil, amlodipine besylate, and atorvastatin calcium hydrate for an extended period. She occasionally self-interrupted her medications, and her type 2 DM was poorly controlled, with hemoglobin A1c levels between 8-9%. She lived with her husband and was generally independent in activities of daily living. Her cognitive function was appropriate for her age, with scores of 26/30 on the Hasegawa's Dementia Scale-Revised (HDS-R), 27/30 on the Mini-Mental State Examination (MMSE), and 20/30 on the Japanese version of the Montreal Cognitive Assessment (MoCA-J). She had a history of smoking 20 cigarettes per day from the age of 20 to 67 years but did not consume alcohol.

The patient was diagnosed with stage IVA squamous cell carcinoma of the lung and liver metastases measuring 14 mm. Treatment was initiated with combined nivolumab (360 mg/body, every three weeks) and ipilimumab (1 mg/kg, every six weeks) therapy starting on day 0. Blood tests before treatment initiation showed normal thyroid hormones but elevated thyroglobulin antibody (TgAb) levels (5.97 IU/mL). After two courses of nivolumab (day 42), blood tests revealed subclinical hyperthyroidism with free thyroxine (F-T4) at 1.74 ng/dl and TSH at 0.03 μIU/mL. Thyroid ultrasonography showed no abnormalities. ICI treatment was continued because this episode was classified as a grade 1 irAE, and thyroid function normalized within a month. CT scans revealed a reduction in the size of both the lung tumor (10 mm) and the liver metastases (9 mm) throughout the treatment course. However, after six courses of nivolumab (day 126), CT scans revealed diffuse ground-glass opacities in both lungs, suggesting drug-induced lung disease, and chemotherapy was discontinued. There were no subjective symptoms, and no requirement for oxygen or steroids was noted.

One month after the last administration, the patient presented in a coma and was transported to a nearby hospital (day 138). Her blood pressure was 43/30 mmHg, and her body temperature was 38.7°C. Arterial blood gas analysis revealed a pH of 7.26, PaCO2 of 36 mmHg, and HCO3- of 11 mmol/L with an anion gap of 25 mEq/L. Blood tests showed elevated WBC counts (9,400/µL), high C-reactive protein (16.30 mg/dl), high creatinine (3.63 mg/dl), high glucose (284 mg/dl), and positive urine ketones. Urine WBC counts were positive, and *Aerococcus urinae* was detected in the urine culture. Although blood cultures were negative, she was diagnosed with sepsis, as indicated by a sequential organ failure assessment (SOFA) score of 11 [7], attributable to a urinary tract infection, along with diabetic ketoacidosis and pre-renal failure. After three days of acute-phase treatment, her blood pressure and creatinine levels normalized. However, despite the continuation of antimicrobial therapy, the fever and tachycardia persisted. The patient was transferred to our hospital on day 143 for further treatment.

Upon admission, she was slightly unconscious (Japan Coma Scale I-1), with a blood pressure of 116/75 mmHg, a heart rate of 139/min regular, a body temperature of 37.9°C, and an SpO2 of 98% on room air. No abnormalities were observed in the chest or abdomen. There was no tenderness or enlargement of the thyroid gland. The blood test results are presented in Table 1.

**Table 1:** Summary of laboratory testing. ACTH: Adrenocorticotropic hormone; LH: Luteinizing hormone; FSH: Follicle-stimulating hormone; PRL: Prolactin; E2: Estradiol; GH: Growth hormone; F-T3: Free triiodothyronine; F-T4: Free thyroxine; TSH: Thyroid-stimulating hormone; TSAb: Thyroid stimulation antibody; TRAb: Thyroid-stimulating hormone receptor antibody; TgAb: Thyroglobulin antibody; TPOAb: Anti-thyroid peroxidase antibody.

Laboratory Test	Values	Reference range
White Blood Cell count (10^3/μL)	5.85	3.3-8.6
Stab cell (%)	2.0	2.0-6.0
Segmented cell (%)	43.0	40.0-75.0
Lymphocyte (%)	33.0	26.0-45.0
Eosinophil (%)	4.0	0.0-6.0
Hemoglobin (g/dL)	13.2	11.6-14.8
Platelet count (10^3/μL)	160	158-348
Aspartate aminotransferase (U/L)	18	13-30
Alanine aminotransferase (U/L)	25	7-23
Alkaline phosphate (U/L)	50	38-113
Lactate dehydrogenase (U/L)	321	124-222
Creatinine kinase (U/L)	135	41-153
Creatinine (mg/dL)	0.47	0.46-0.79
C-reactive protein (mg/dL)	4.66	<0.14
Sodium (mEq/L)	139	138-145
Potassium (mEq/L)	3.5	3.6-4.8
Chloride (mEq/L)	101	101-108
Corrected Calcium (mg/dL)	8.7	8.8-10.1
Triglyceride (mg/dL)	178	30-117
Total cholesterol (mg/dL)	121	142-248
High Density Lipoprotein Cholesterol (mg/dL)	12	48-103
Low Density Lipoprotein Cholesterol (mg/dL)	73	65-163
Casual glucose (mg/dL) 11:00h	253	<200
Hemoglobin A1c (%)	10.0	4.9-6.0
C-peptide immunoreactivity (ng/mL)	3.1	1.10-3.30
Anti-GAD antibody (U/mL)	<5.0	<5.0
Cortisol (μg/dL) 11:00 h	2.2	6.4-21.0
ACTH (pg/mL)	<1.5	7.2-63.3
LH (mlU/mL)	15.49	5.7-64.3 (post menopause)
FSH (mlU/mL)	25.34	<157.8 (post menopause)
PRL (ng/mL)	13.36	6.12-30.54
E2 (pg/mL)	19	<47 (post menopause)
Progesterone (ng/mL)	<0.1	<0.33 (post menopause)
GH (ng/mL)	0.59	0.13-9.88
F-T3 (pg/mL)	10.11	2.10-3.80
F-T4 (ng/dL)	5.89	0.82-1.63
TSH (μIU/mL)	0.01	0.38-4.31
TSAb (%)	96	<110
TRAb (IU/L)	<0.8	<2.0
TgAb (IU/mL)	17.0	<4.11
TPOAb (IU/mL)	2.01	<5.61
Aldosterone (pg/mL)	32.8	4.0-82.1
Renin (ng/mL/hr)	2.3	2.21-39.49

**Figure 1:**
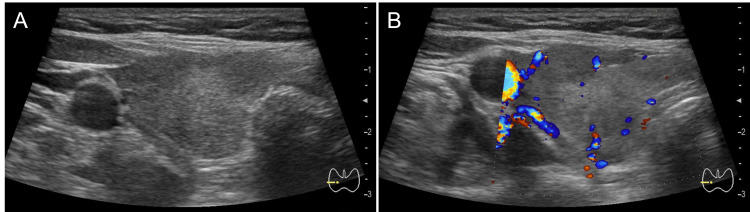
An ultrasound of right thyroid lobe. (A) Ultrasound of the right thyroid lobe showing a heterogeneous thyroid gland with a hypoechoic area. (B) Ultrasound of the right thyroid lobe showing decreased Doppler flow.

Free triiodothyronine (F-T3) was 10.11 pg/ml, F-T4 was 5.9 ng/dl, TSH was 0.01 μIU/ml, adrenocorticotrophic hormone (ACTH) was < 1.5 pg/ml, and cortisol (at 11:00 h) was 2.2 μg/dl, while other pituitary hormones, renin, and aldosterone were within the normal range. The fractions in the WBCs showed non-suppressed eosinophils. Treatment for thyrotoxicosis was initiated with potassium iodide (50 mg/day) and bisoprolol fumarate (2.5 mg/day). Thyroid ultrasonography revealed heterogeneous parenchyma with a hypoechoic area and decreased Doppler flow, incompatible with Graves' disease (Figure 1).

Blood tests were positive for TgAb and negative for TSH receptor antibody (TRAb). The diagnosis of painless thyroiditis was confirmed based on the absence of tenderness and enlargement of the thyroid gland, the presence of thyrotoxicosis, decreased Doppler flow, positive TgAb, and negative TRAb results. Potassium iodide was discontinued on day 146.

**Figure 2:**
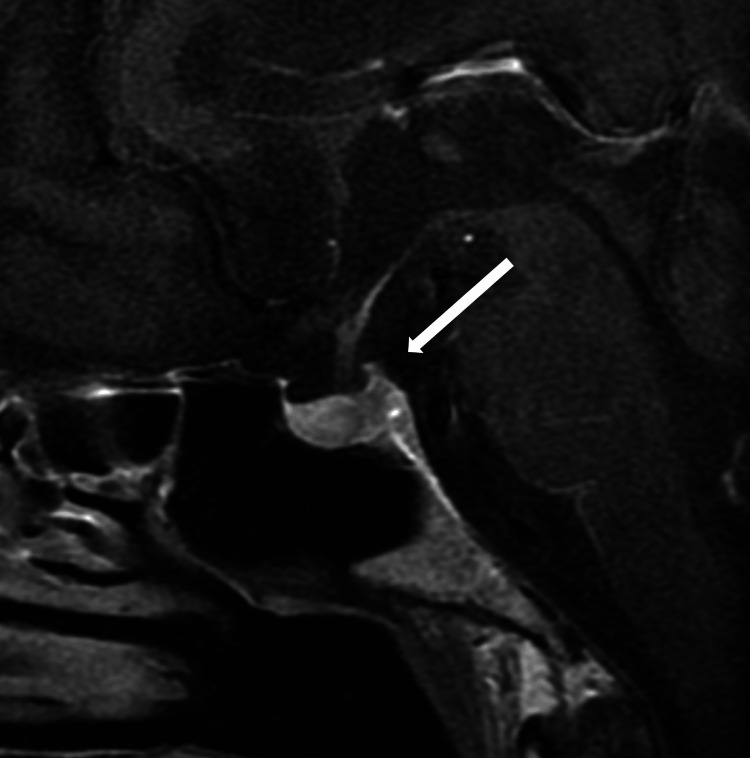
A T1-weighted sagittal image of the pituitary on MRI. Pituitary MRI showed no noticeable change.

Low ACTH and cortisol levels indicated secondary adrenal insufficiency, and IV hydrocortisone (200 mg/day for one day, 150 mg/day for two days, 75 mg/day for two days) was initiated. Hydrocortisone was later tapered and transitioned to oral medication (15 mg/day; 10 mg in the morning and 5 mg in the evening) as the patient’s condition improved. MRI of the pituitary gland on day 151 revealed no abnormalities (Figure 2).

The corticotropin-releasing hormone (CRH) loading test on day 157 showed a low response of both ACTH and cortisol (Table 2).

**Table 2:** CRH loading test. CRH loading test showed low ACTH and cortisol response. CRH: Corticotropin-releasing hormone; ACTH: Adrenocorticotropic hormone.

Minutes after stimulation	0	30	60	90
Cortisol (μg/dL)	0.8	0.7	0.7	0.8
ACTH (pg/mL)	<1.5	<1.5	<1.5	<1.5

The ACTH loading test on day 158 revealed a low cortisol response with a peak cortisol level of less than 14 μg/ml (Table 3) [8].

**Table 3:** ACTH loading test. ACTH loading test showed low cortisol response. ACTH: Adrenocorticotropic hormone.

Minutes after stimulation	0	30	60
Cortisol (μg/dL)	0.8	5.8	8.1

Growth hormone-releasing peptide-2 (GHRP-2) and luteinizing hormone-releasing hormone loading tests showed normal responses. ACTH and cortisol levels remained low (ACTH < 1.5 pg/ml and cortisol 4.5 μg/dl on day 181).

Regarding DM, ketoacidosis improved upon admission. The anti-glutamic acid decarboxylase (GAD) antibody was negative, and insulin secretion was sufficient (C-peptide 3.1 ng/ml), indicating type 2 diabetes. She was treated with intensive insulin therapy, followed by oral diabetic medications.

The patient's general condition improved, and she was transferred to a rehabilitation hospital on day 183. Upon discharge, her F-T3 (2.80 pg/ml) and F-T4 (1.68 ng/dl) levels had decreased, although her TSH remained undetectable.

## Discussion

We encountered a rare case of thyrotoxicosis caused by painless thyroiditis and secondary adrenal insufficiency (AI) occurring simultaneously after the combined use of two ICIs; nivolumab, an anti-PD-1 antibody, and ipilimumab, an anti-CTLA-4 antibody.

Because of its efficiency in the survival of patients with malignant tumors in various organs, ICIs have become a standard therapeutic option for patients with inoperable tumors [1]. However, the use of ICIs can induce severe side effects, such as endocrine irAEs [2]. Among the endocrine irAEs, thyroid dysfunction accounts for 35-42% [9, 10]. The most common cause of thyroid dysfunction is destructive thyroiditis, whereas Graves’ disease is quite rare [11]. Thyrotoxicosis accounts for 12% of endocrine irAEs [9] and is reported to be more frequently caused by anti-PD-1 antibodies than by anti-CTLA-4 antibodies [3]. Moreover, the combined use of both antibodies is approximately 3.3 times more likely to cause thyrotoxicosis than the use of anti-PD-1 antibodies alone [9]. Additionally, the presence of thyroid autoantibodies is estimated to increase the risk of ICI-induced thyrotoxicosis. Okada N et al. reported that patients positive for anti-thyroid antibodies (TgAb and/or anti-thyroid peroxidase antibody) before anti-PD-1 antibody treatment had a higher incidence of thyroid dysfunction (34.1%) than those negative for anti-thyroid antibodies (2.4%) [12]. Iwama S et al. reported that the incidence of thyroid dysfunction in patients treated with anti-PD-1/CTLA-4 antibody combination therapy was 60.0% in antibody-positive patients and 23.5% in antibody-negative patients [13]. Thus, in this case, the concomitant use of ICI and the presence of an anti-thyroid antibody may have led to thyrotoxicosis. Furthermore, the average age of onset for thyrotoxicosis caused by irAEs is reported to be 60 years [9], while our patient was 80 years old, which is significantly higher. The development of thyroid dysfunction is associated with better outcomes after treatment with ICIs in non-small cell lung carcinoma [14].

In our case, the decreased ACTH level and the low response to the CRH stimulation test indicated that the AI was due to hypopituitarism. It has been reported that the most common form of AI in irAEs is secondary, mainly caused by hypopituitarism [15]. The major cause of hypopituitarism is hypophysitis, whose incidence increases to 6.4% with the use of anti-PD-1/CTLA-4 antibody combination, compared to 3.2% with anti-CTLA-4 antibodies alone, and 0.4% with anti-PD-1 antibodies [3]. Among pituitary dysfunctions, the incidences of secondary AI, secondary hypothyroidism, and secondary hypogonadism are 6.1%, 7.6%, and 7.5%, respectively [16].

Although the exact mechanism of CTLA-4 or PD-1 antibody-induced hypophysitis remains unclear, activation of antibody-dependent cell-mediated cytotoxicity (ADCC) and the complement system may play a role in anti-CTLA-4-induced hypophysitis in a mouse model [11]. Ectopic expression of CTLA-4 in the pituitary and activated effector and regulatory T cells were observed in this model [6]. Since ipilimumab is an IgG1 class antibody, which could more strongly activate both ADCC and the classical complement pathway, this class of antibody may contribute to the higher incidence of hypophysitis compared to other ICIs. Although anti-pituitary antibodies were present in patients with ipilimumab-related hypophysitis [17], the involvement of ADCC and complement activation has not been clarified in anti-CTLA-4-induced hypophysitis in humans.

Anti-CTLA-4-induced hypophysitis typically shows MRI abnormalities (98%), characterized by enlargement of the pituitary gland [18] and multiple anterior pituitary hormone deficiencies [19]. Anti-PD-1-induced hypophysitis has a lower rate of MRI abnormalities (28%) [18], with isolated ACTH deficiency being the most prevalent occurrence (97%) [20]. In ipilimumab and nivolumab combination therapy, enlargement of the pituitary gland is observed in most cases (94%) [18]. In this case, however, the MRI did not show any notable changes in the pituitary gland, which is atypical for nivolumab/ipilimumab-induced hypopituitarism. It is also reported that pituitary gland enlargement may not be observed if ACTH secretion is the only impairment involved [15].

Spontaneous resolution is rarely reported in ICI-induced hypophysitis, and long-term hormonal replacement is required in most cases [6]. Although the fatality rate of ICI-induced hypophysitis and secondary AI is unknown, pituitary dysfunction induced by ICIs is associated with a better prognosis in non-small cell lung carcinoma [14].

Although there have been several reports of multiple endocrine irAEs caused by ICI combination therapy, only four cases of AI and thyrotoxicosis due to nivolumab and ipilimumab therapy have been reported (Table 4) [21-24].

**Table 4:** Previously reported cases of thyrotoxicosis and adrenal insufficiency caused by nivolumab and ipilimumab. CR: Complete remission; Ipi: Ipilimumab; Niv: Nivolumab; PG: Plasma glucose; PR: Partial remission; Sun: Sunitinib; T1D: Type 1 diabetes; NA: Not available. *Duration after the first administration of ICIs; **Outcome of malignancies.

Case (Ref)	Age and Sex	Malignancy	ICIs	irAE	Duration to the appearance of irAE*	Treatment for irAE	Outcome**	Other complications
Lowe JR et al. [21]	54 M	Cutaneous melanoma	Ipi+Niv	Thyrotoxicosis	2 weeks	Prednisone metoprolol levothyroxine hydrocortisone insulin	CR	Skin rash, autoimmune hepatitis, colitis
				→Hypothyroidism	9 weeks			
				Hypophysitis	4 months			
				Fulminant T1D	4 months			
Newman C et al. [22]	46 F	Malignant melanoma	Ipi+Niv	Thyrotoxicosis	24 weeks	Beta blocker hydrocortisone	CR	Hypercalcemia, acute pancreatitis
				Hypophysitis	30 weeks			
Hino C et al. [23]	45 M	Sarcomatoid renal cell carcinoma	Ipi+Niv →Niv →Sun →Niv	Thyrotoxicosis	70 days	Levothyroxine insulin hydrocortisone	CR	Acute interstitial nephritis
				→Hypothyroidism	112 days			
				T1D	171 days			
				Hypophysitis	239 days			
Iesaka H et al. [24]	81 M	Renal cell carcinoma	Ipi+Niv	Thyrotoxicosis	2 course	Levothyroxine insulin hydrocortisone	N.A.	None
				→Hypothyroidism	83 days			
				Fulminant T1D	75 days			
				Hypophysitis	89 days			
Our case	80 F	Lung carcinoma	Ipi+Niv	Thyrotoxicosis	143 days	Beta blocker iodine hydrocortisone	PR	Drug-induced lung disease
				Hypophysitis	143 days			

Previous reports indicate that the duration before the onset of hypophysitis and thyrotoxicosis caused by a combination of anti-PD-1 and anti-CTLA-4 antibodies differs, averaging 10.3 weeks and two weeks after the first administration, respectively [4-6]. Our case presented secondary AI and thyrotoxicosis of late onset, observed five months after the first administration of nivolumab and ipilimumab. It is noteworthy that in previous cases of both AI and thyrotoxicosis induced by nivolumab and ipilimumab (Table 4), the time of onset was delayed, similar to that in our case. However, in all those cases, thyrotoxicosis preceded hypophysitis, making the simultaneous onset in our case unique.

Moreover, subclinical hyperthyroidism occurred 42 days after the first administration during ongoing combination treatment, which is consistent with previous reports. It is known that in some patients, painless thyroiditis can relapse several times, especially in males and in those with younger onset [25]. Even though the abnormal thyroid function in this case spontaneously returned to normal levels, thyroiditis recurred, with the onset overlapping that of the AI, and the level of thyroiditis reaching thyrotoxicosis. In this context, although most frequently the first event of thyroiditis precedes that of hypophysitis [26], careful follow-up of thyroid function is necessary, especially if the first event is not severe enough to discontinue the medication. It is unclear, however, whether thyrotoxicosis could have been avoided if we had discontinued ICIs at the first episode of subclinical hyperthyroidism. Since thyroid hormones accelerate the clearance of steroid hormones [27], the simultaneous onset of AI and thyrotoxicosis could worsen steroid hormone deficiency, leading to a life-threatening condition. When thyrotoxicosis is combined with AI, adequate steroid replacement should be administered [28]. Although we were unable to follow up on this case because the patient was transferred to a different hospital, painless thyroiditis may lead to hypothyroidism in the long term. If the patient develops hypothyroidism, thyroid hormones should be appropriately replaced. In this case, the patient also suffered from diabetic ketoacidosis (DKA). Since she originally had type 2 DM, it is estimated that a severe infection triggered the DKA without the onset of type 1 DM, which is known to be another endocrinological irAE. The incidence of type 1 DM after ICI use is 0.2% [3], and most of the reported cases of type 1 DM are associated with anti-PD-1 antibodies, with DKA occurring in 71% of type 1 DM cases [29]. After starting ICI treatment, frequent blood glucose monitoring is essential, and when blood glucose rises, insulin treatment should be promptly initiated along with checking insulin secretion capacity and anti-islet cell antibodies.

IrAEs occurring after the discontinuation of immunotherapy are common. Some cases of irAE are observed even more than six months after discontinuation [30]. This indicates that careful follow-up of the incidence of irAEs is necessary even after ICI discontinuation. In our case, irAEs occurred only one month after the last administration, which means that the patient was still at high risk for recurrence after discontinuation.

## Conclusions

Combination treatment with ICIs could increase the risk for multiple irAEs involving endocrine organs. This case emphasizes that thyrotoxicosis and AI can occur simultaneously after combination treatment with ICIs. Careful follow-up and early detection of endocrine disorders are critical in patients treated with combination therapy involving ICIs.

## References

[1] Shiravand Y, Khodadadi F, Kashani SM (2022). Immune checkpoint inhibitors in cancer therapy. Curr Oncol.

[2] Iwama S, Kobayashi T, Arima H (2021). Clinical characteristics, management, and potential biomarkers of endocrine dysfunction induced by immune checkpoint inhibitors. Endocrinol Metab (Seoul).

[3] Barroso-Sousa R, Barry WT, Garrido-Castro AC, Hodi FS, Min L, Krop IE, Tolaney SM (2018). Incidence of endocrine dysfunction following the use of different immune checkpoint inhibitor regimens: a systematic review and meta-analysis. JAMA Oncol.

[4] Bai X, Chen X, Wu X, Huang Y, Zhuang Y, Lin X (2020). Immune checkpoint inhibitor-associated thyroid dysfunction: a disproportionality analysis using the WHO Adverse Drug Reaction Database, VigiBase. Eur J Endocrinol.

[5] Iyer PC, Cabanillas ME, Waguespack SG (2018). Immune-related thyroiditis with immune checkpoint inhibitors. Thyroid.

[6] Di Dalmazi G, Ippolito S, Lupi I, Caturegli P (2019). Hypophysitis induced by immune checkpoint inhibitors: a 10-year assessment. Expert Rev Endocrinol Metab.

[7] Singer M, Deutschman CS, Seymour CW (2016). The third international consensus definitions for sepsis and septic shock (Sepsis-3). JAMA.

[8] Javorsky BR, Raff H, Carroll TB, Algeciras-Schimnich A, Singh RJ, Colón-Franco JM, Findling JW (2021). New cutoffs for the biochemical diagnosis of adrenal insufficiency after ACTH stimulation using specific cortisol assays. J Endocr Soc.

[9] Muir CA, Clifton-Bligh RJ, Long GV (2021). Thyroid immune-related adverse events following immune checkpoint inhibitor treatment. J Clin Endocrinol Metab.

[10] Patel NS, Oury A, Daniels GA (2018). Incidence of thyroid function test abnormalities in patients receiving immune-checkpoint inhibitors for cancer treatment. Oncologist.

[11] Chang LS, Barroso-Sousa R, Tolaney SM, Hodi FS, Kaiser UB, Min L (2019). Endocrine toxicity of cancer immunotherapy targeting immune checkpoints. Endocr Rev.

[12] Okada N, Iwama S, Okuji T (2020). Anti-thyroid antibodies and thyroid echo pattern at baseline as risk factors for thyroid dysfunction induced by anti-programmed cell death-1 antibodies: a prospective study. Br J Cancer.

[13] Iwama S, Kobayashi T, Yasuda Y (2022). Increased risk of thyroid dysfunction by PD-1 and CTLA-4 blockade in patients without thyroid autoantibodies at baseline. J Clin Endocrinol Metab.

[14] Kobayashi T, Iwama S, Yasuda Y (2020). Pituitary dysfunction induced by immune checkpoint inhibitors is associated with better overall survival in both malignant melanoma and non-small cell lung carcinoma: a prospective study. J Immunother Cancer.

[15] Okura N, Asano M, Uchino J (2020). Endocrinopathies associated with immune checkpoint inhibitor cancer treatment: a review. J Clin Med.

[16] Byun DJ, Wolchok JD, Rosenberg LM, Girotra M (2017). Cancer immunotherapy - immune checkpoint blockade and associated endocrinopathies. Nat Rev Endocrinol.

[17] Iwama S, De Remigis A, Callahan MK, Slovin SF, Wolchok JD, Caturegli P (2014). Pituitary expression of CTLA-4 mediates hypophysitis secondary to administration of CTLA-4 blocking antibody. Sci Transl Med.

[18] Faje A, Reynolds K, Zubiri L (2019). Hypophysitis secondary to nivolumab and pembrolizumab is a clinical entity distinct from ipilimumab-associated hypophysitis. Eur J Endocrinol.

[19] Atkins P, Ur E (2020). Primary and ipilimumab-induced hypophysitis: a single-center case series. Endocr Res.

[20] Husebye ES, Castinetti F, Criseno S (2022). Endocrine-related adverse conditions in patients receiving immune checkpoint inhibition: an ESE clinical practice guideline. Eur J Endocrinol.

[21] Lowe JR, Perry DJ, Salama AK, Mathews CE, Moss LG, Hanks BA (2016). Genetic risk analysis of a patient with fulminant autoimmune type 1 diabetes mellitus secondary to combination ipilimumab and nivolumab immunotherapy. J Immunother Cancer.

[22] Newman C, Kgosidalwa O, Hakami OA, Kennedy C, Grogan L, Agha A (2021). Multiple endocrinopathies, hypercalcaemia and pancreatitis following combined immune checkpoint inhibitor use- case report and review of literature. BMC Endocr Disord.

[23] Hino C, Nishino K, Pham B, Jeon WJ, Nguyen M, Cao H (2022). Nivolumab plus ipilimumab induced endocrinopathy and acute interstitial nephritis in metastatic sarcomatoid renal-cell carcinoma: a case report and review of literature. Front Immunol.

[24] Iesaka H, Kameda H, Miya A (2023). Fulminant ACTH decrease following diabetic ketoacidosis induced by immune checkpoint inhibitor combination therapy with nivolumab and ipilimumab: a case report. Medicine (Baltimore).

[25] Nishimaki M, Isozaki O, Yoshihara A, Okubo Y, Takano K (2009). Clinical characteristics of frequently recurring painless thyroiditis: contributions of higher thyroid hormone levels, younger onset, male gender, presence of thyroid autoantibody and absence of goiter to repeated recurrence. Endocr J.

[26] Levy M, Abeillon J, Borson-Chazot F (2019). Immune checkpoint inhibitors therapy-induced hypophysitis is frequently associated with previous thyroid disorders: results from ImmuCare study. Endocr Abstr.

[27] Jubiz W, Meikle AW (1979). Alterations of glucocorticoid actions by other drugs and disease states. Drugs.

[28] Newrick PG (1984). Addison's disease and thyrotoxicosis presenting simultaneously. Postgrad Med J.

[29] de Filette JM, Pen JJ, Decoster L (2019). Immune checkpoint inhibitors and type 1 diabetes mellitus: a case report and systematic review. Eur J Endocrinol.

[30] Couey MA, Bell RB, Patel AA (2019). Delayed immune-related events (DIRE) after discontinuation of immunotherapy: diagnostic hazard of autoimmunity at a distance. J Immunother Cancer.

